# Glucosamine for knee osteoarthritis: an umbrella review

**DOI:** 10.3389/fnut.2026.1806413

**Published:** 2026-08-11

**Authors:** Yanfei Li, Ruixin Wang, Jia Xu, Yun Shen, Gang Hu, Shengdi Lu

**Affiliations:** 1 Department of Rehabilitation Medicine, Huashan Hospital, Shanghai, China; 2 School of Public Health, Fudan University, Shanghai, China; 3 Pennington Biomedical Research Center, Baton Rouge, LA, United States; 4 Department of Orthopaedics, Shanghai Sixth People’s Hospital, Shanghai, China

**Keywords:** glucosamine, joint space narrowing, knee osteoarthritis, meta-analysis, pain, umbrella review

## Abstract

**Background:**

Knee osteoarthritis (OA) is a common cause of pain and disability without a cure. Glucosamine is widely used as a symptomatic treatment owing to its safety, yet its clinical efficacy remains uncertain amid mixed trial results and conflicting guidelines. This umbrella review synthesized evidence from published systematic reviews and meta-analyses on the effectiveness and safety of glucosamine in knee OA.

**Methods:**

We searched MEDLINE (via PubMed), Embase, Web of Science, and the Cochrane Library up to November 2025. Eligible studies were systematic reviews or meta-analyses of randomized trials in adults with knee OA comparing glucosamine (any formulation) with placebo or other non-surgical comparators and reporting pain, physical function, or joint structure. Two reviewers independently screened, extracted data, and assessed quality (AMSTAR-2). Key outcomes were pain (visual analog scale [VAS] and WOMAC [Western Ontario and McMaster Universities Osteoarthritis Index]), functional measures, joint space narrowing (JSN), and adverse events. Overlap among shared primary trials was quantified using the corrected covered area, and the resulting dependence was addressed using robust variance estimation to pool standardized mean differences (SMDs), with *p* < 0.05 considered significant. Effect sizes were interpreted against minimal clinically important difference (MCID) thresholds.

**Results:**

Nineteen reviews were included (most low or critically low quality). Glucosamine produced a small but statistically significant reduction in VAS pain (SMD − 0.36, *p* = 0.02), whereas WOMAC pain showed no significant difference. Functional outcomes (WOMAC total and function) showed no significant improvement. Glucosamine was associated with significant slowing of JSN progression (SMD − 0.32, *p* = 0.01), though this signal derived mainly from long-duration sulfate trials. The magnitude of the significant effects was at or below accepted MCID thresholds. Adverse event rates did not differ from placebo.

**Conclusion:**

Glucosamine demonstrated at most minimal-to-modest efficacy for knee OA, yielding a small pain reduction and possible slowing of radiographic progression but no clear functional benefit, with effects at or below the threshold of clinical importance. Given the low quality of evidence, extensive overlap among reviews, and the predominantly out-of-pocket nature of glucosamine use, these findings should be interpreted with caution and weighed against cost in shared decision-making.

**Systematic review registration:**

https://www.crd.york.ac.uk/prospero/, identifier (CRD420251207513).

## 1 Background

Osteoarthritis (OA) is a highly prevalent joint disease and a leading cause of disability, especially in older adults. Knee OA is the most common form; Global Burden of Disease estimates place the age-standardised prevalence of knee OA at approximately 3.8% of the population, corresponding to several hundred million people affected worldwide (1–3). It is characterized by cartilage degeneration, inflammation, pain and stiffness that can severely impair mobility and quality of life. There is currently no cure or disease-modifying therapy for OA (4); management focuses on relieving symptoms and preserving function. Nonpharmacologic strategies, including patient education, weight control, exercise and physical therapy, are fundamental. Among medications, topical and oral nonsteroidal anti-inflammatory drugs are often first-line for pain control. Slow-acting ‘symptomatic’ agents (SYSADOAs) such as glucosamine and chondroitin are also commonly used as background therapy in knee OA (4).

Glucosamine is a natural amino sugar present in cartilage and is available in various formulations. Over-the-counter supplements usually contain glucosamine hydrochloride, while prescription crystalline glucosamine sulfate (typically 1,500 mg/day) is used in many clinical studies (4). Proponents suggest glucosamine may support cartilage health and have anti-inflammatory effects. Because of its safety profile and widespread availability, glucosamine is one of the most popular supplements for OA worldwide, and surveys of clinical practice indicate that a substantial proportion of practitioners continue to recommend it for OA symptoms despite divergent guideline positions (5). Nonetheless, glucosamine’s clinical efficacy remains controversial.

Evidence from individual randomized controlled trials and from their syntheses has been inconsistent, with positive and null findings reported across the literature (6–8). Some systematic reviews report modest benefits of glucosamine for knee pain and function. For instance, a 2023 review found that glucosamine significantly reduced global knee pain versus placebo (6). However, the effect sizes were small and improvements in stiffness or function were minimal. By contrast, a large network meta-analysis (7) found that glucosamine (alone or with chondroitin) produced no clinically meaningful improvement in pain or joint space narrowing compared to placebo. Consistently, high-quality trials with low bias often show little difference between glucosamine and placebo. A Cochrane review noted that pooled analyses modestly favored glucosamine (e.g., 22% pain improvement overall), but restricted analyses of rigorously conducted trials found no significant benefit on standard pain or function scales (6, 7). Importantly, these analyses indicate that glucosamine is generally well tolerated, with adverse event rates similar to placebo.

The uncertainty in the literature is reflected in clinical guidelines and practice. Major osteoarthritis guidelines diverge on glucosamine: the ESCEO (European) panel strongly recommends prescription glucosamine sulfate for knee OA, while groups like Osteoarthritis Research Society International (OARSI) and the American College of Rheumatology advise against its use, and American Academy of Orthopaedic Surgeons (AAOS) gives only a weak endorsement (9–13). This split likely arises from differing interpretations of the inconsistent trial results. In practice, many physicians continue to advise glucosamine use for knee OA despite guideline ambivalence (5). Patients and providers thus face conflicting messages about the value of glucosamine.

Given the volume of studies and reviews on glucosamine, and the ongoing debate about its efficacy, a comprehensive synthesis is needed. The purpose of the current umbrella review is to collate and evaluate evidence from all published systematic reviews and meta-analyses on glucosamine in knee OA. We will compare glucosamine (usually 1,500 mg/day) against placebo and other interventions (e.g., exercise, NSAIDs, intra-articular therapies, education/lifestyle) across key outcomes: pain, strength, physical function, patient-reported measures, and metabolic or organ-system effects (such as glycemic and cardiovascular parameters). The study aims to assess the overall credibility and strength of evidence for glucosamine’s benefits and risks. We hypothesize that the aggregate evidence will show at most modest symptomatic benefit of glucosamine, with little impact on disease progression or metabolic outcomes, and no significant safety concerns beyond placebo.

## 2 Methods

### 2.1 Registration and protocol

This study was conducted as an umbrella meta-analysis, synthesizing evidence from published systematic reviews and meta-analyses to evaluate the effectiveness of glucosamine on pain, functional, and structural outcomes in knee osteoarthritis. The methodology followed established recommendations for umbrella reviews (14), and the reporting adhered to the PRISMA 2020 guidelines for systematic reviews of reviews (15). This umbrella review was prospectively registered in the International Prospective Register of Systematic Reviews (PROSPERO; registration number CRD420251207513).

### 2.2 Information sources and search strategy

A comprehensive literature search was conducted to evaluate the clinical effectiveness of glucosamine supplementation on pain, functional outcomes, and structural progression in patients with OA. Relevant studies were identified through systematic searches of MEDLINE (via PubMed), Embase, Web of Science, and the Cochrane Database of Systematic Reviews from inception to November 25, 2025. The search strategy combined Medical Subject Headings (MeSH) and free-text terms related to knee osteoarthritis, glucosamine, and evidence synthesis. Key search terms included ‘knee osteoarthritis,’ ‘osteoarthritis,’ ‘glucosamine, ‘glucosamine sulfate,’ ‘glucosamine hydrochloride,’ ‘systematic review,’ and ‘meta-analysis.’ Boolean operators and database-specific syntax were applied to ensure comprehensive retrieval. The full search strategy is provided in the Supplementary materials.

### 2.3 Inclusion and exclusion criteria

#### 2.3.1 Inclusion criteria

Studies were considered eligible for inclusion if they met all of the following criteria. First, studies had to be systematic reviews or meta-analyses that included adult patients (≥18 years) diagnosed with knee osteoarthritis, based on clinical and/or radiographic criteria, with no restrictions on disease severity, sex, or geographic location. Second, the reviews needed to evaluate glucosamine-based interventions, administered via any route (including oral, intra-articular, topical, or combination approaches), either as monotherapy or in combination with other non-surgical interventions, provided that the effects attributable to glucosamine could be independently extracted or clearly distinguished. Third, eligible reviews were required to synthesize evidence from RCTs or observational studies using a systematic review, meta-analysis, or network meta-analysis framework. For network meta-analyses, only reviews reporting direct or indirect pairwise comparisons involving glucosamine versus other non-surgical interventions were included. When quantitative effect estimates were not available, findings were retained for qualitative synthesis only. Finally, only studies published in English in peer-reviewed journals were included.

#### 2.3.2 Exclusion criteria

Studies were excluded if they met any of the following criteria. First, where two or more publications represented the same systematic review—for example, an exact duplicate publication, a conference abstract of a subsequently published review, or an earlier version superseded by an updated review (such as a prior edition of the same Cochrane review)—only the single most complete and up-to-date version was retained, defined *a priori* as the publication with the largest number of relevant primary trials and the higher AMSTAR-2 rating. This criterion was applied only to remove redundant reports of the same review; reviews that were methodologically independent but happened to share some of the same underlying primary trials were all retained, and the resulting overlap of primary trials across the included reviews was instead handled analytically using the corrected covered area and robust variance estimation, as described in the Statistical Analysis section. Second, reviews were excluded if they did not report extractable quantitative effect estimates for at least one outcome of interest, operationalized as the absence of both (i) a point estimate (mean difference, standardized mean difference, or risk ratio) and (ii) a corresponding measure of precision (95% confidence interval, standard error, or variance) that would permit conversion to a standardized mean difference. Third, reviews were excluded if they did not report the search strategy, eligibility criteria, and method of evidence synthesis in sufficient detail to permit replication, or if they did not include any formal assessment of the risk of bias or methodological quality of their primary studies. These thresholds were applied independently by two reviewers, and discrepancies were resolved by discussion or by a third reviewer.

### 2.4 Data extraction and handling

Data extraction was independently conducted by two reviewers using a standardized form. Extracted information included author and publication year, characteristics of included primary studies, type of glucosamine intervention and comparator, outcome measures, and pooled effect estimates with corresponding 95% confidence intervals (16).

Outcomes of interest comprised pain outcomes (VAS, WOMAC pain), functional outcomes (WOMAC function and WOMAC total score), and structural outcomes (joint space narrowing, JSN). When multiple outcomes or time points were reported, the most commonly reported or longest follow-up data were preferentially extracted. For each review, we also recorded, where reported, the glucosamine salt formulation (sulfate versus hydrochloride), the daily dose and regimen, and the treatment duration of the contributing primary trials, so that the predominant interventions could be characterized and outcomes could be examined descriptively by formulation. Across the included reviews, the most frequently evaluated regimen was oral glucosamine sulfate 1,500 mg/day; however, dose, frequency, and treatment duration were inconsistently and incompletely reported at the review level, which precluded formal dose- or duration-stratified pooling in the robust variance estimation model.

Effect estimates were harmonized as standardized mean differences (SMDs). When necessary, standard errors and variances were derived from reported confidence intervals. To identify and quantify the degree of overlap among the primary trials shared across the included reviews, we constructed a citation matrix mapping each primary randomized controlled trial to every review in which it appeared and calculated the corrected covered area (CCA), with CCA values of <5%, 6–10%, 11–15, and >15% interpreted as slight, moderate, high, and very high overlap, respectively (17). Because this matrix confirmed substantial overlap of primary trials across reviews, pooled analyses were subsequently performed using robust variance estimation (RVE) to account for the resulting statistical dependence among effect sizes (18). Disagreements in data extraction were resolved through discussion or consultation with a third reviewer (16).

### 2.5 Statistical analysis

All statistical analyses were performed using R software (version 4.4.0). Effect estimates extracted from eligible meta-analyses were synthesized using SMDs with corresponding 95% confidence intervals (CIs). Negative SMD values indicated improvement in pain or functional outcomes and reduced progression for structural outcomes. Robust variance estimation was implemented using the robumeta package, and data manipulation and visualization were conducted using the dplyr, readxl, and ggplot2 packages (19). Given the substantial overlap of primary studies and the resulting correlated effect sizes across the included meta-analyses, pooled analyses were conducted using robust variance estimation (RVE) with a correlated-effects model, which yields valid standard errors even when the exact covariance structure among dependent effect sizes is unknown (18). The within-study correlation parameter (*ρ*) was set to 0.8, and the Tipton small-sample correction was applied to obtain adjusted standard errors, confidence intervals, and *p* values (19). A sensitivity analysis varying ρ from 0.6 to 1.0 was performed to confirm that the pooled estimates were not materially affected by the choice of this parameter. Statistical significance was defined as a two-sided p value < 0.05. Effect sizes were interpreted using the conventional Cohen benchmarks (approximately 0.2, 0.5, and 0.8 for small, moderate, and large effects, respectively), and were additionally contextualized against published minimal clinically important difference (MCID) thresholds for knee osteoarthritis outcomes (20–23). Results were summarized in tabular form, and RVE-based forest plots for each outcome were generated and presented in the Supplementary materials.

### 2.6 Risk of bias assessment and methodological quality (AMSTAR-2)

The methodological quality of the included systematic reviews and meta-analyses was assessed using the AMSTAR-2 tool (24). This instrument evaluates key domains related to the design, conduct, and reporting of systematic reviews, including protocol registration, literature search strategy, risk of bias assessment, and appropriateness of statistical methods.

Each review was independently assessed by two reviewers and classified as having high, moderate, low, or critically low methodological quality according to established AMSTAR-2 criteria. Disagreements were resolved through discussion or consultation with a third reviewer.

The quality assessments were used to inform the interpretation of findings and the assessment of the overall strength of evidence but were not applied as exclusion criteria in the synthesis.

## 3 Results

### 3.1 Literature retrieval

**Figure 1:**
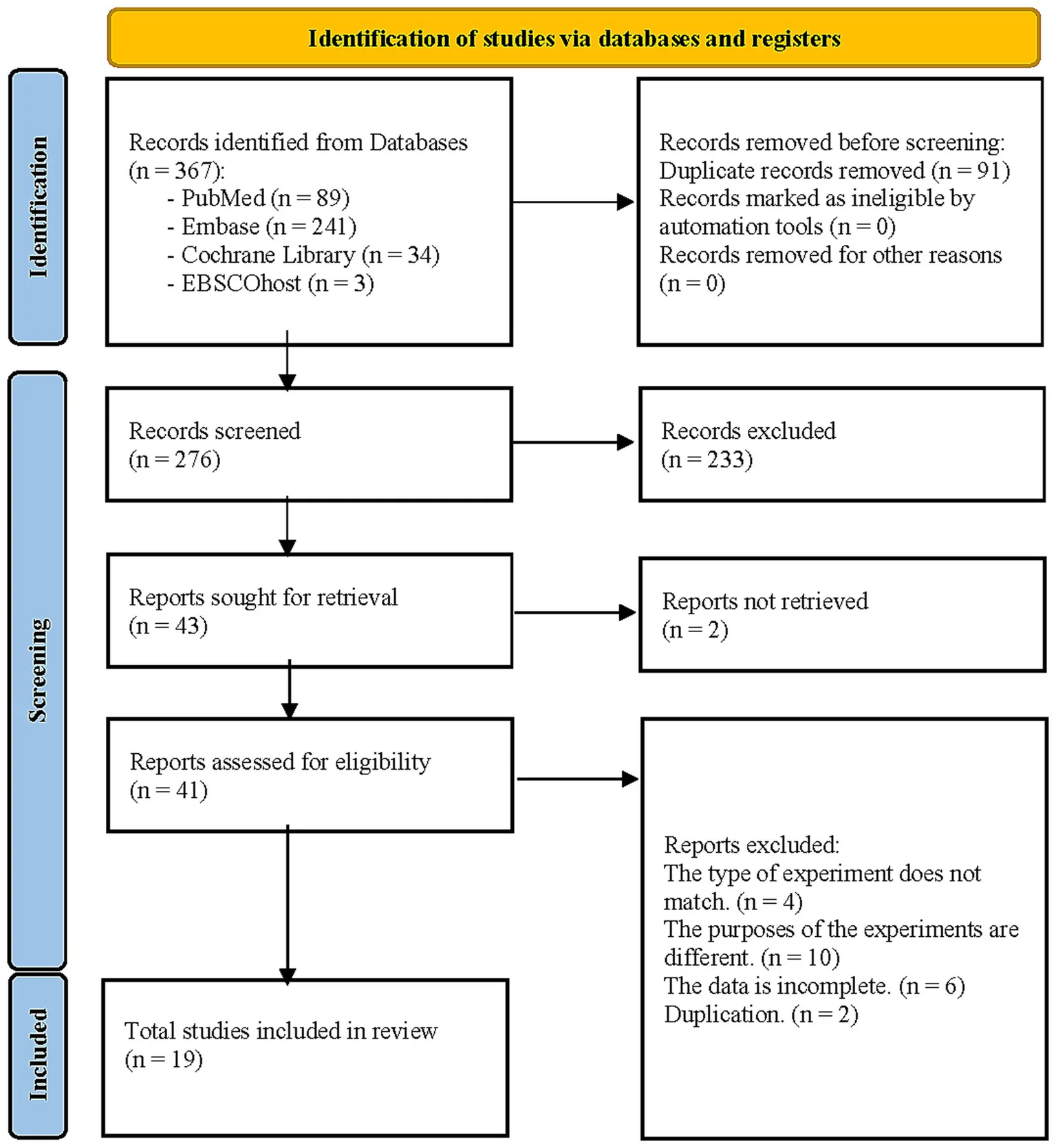
The flow chart of included studies.

The PRISMA 2020 flow diagram is presented in Figure 1. A total of 367 records were identified through a systematic literature search, and after stepwise screening, 19 systematic reviews and meta-analyses were included in the synthesis (6, 25–42). One further publication initially retrieved as a systematic review (4) was, on full-text assessment, an umbrella review of glucosamine sulfate; it was therefore not counted among the included primary syntheses and was instead used separately as an external reference point for cross-validation of our findings. The included studies evaluated the effects of glucosamine on pain, functional, and structural outcomes in patients with knee osteoarthritis, using measures such as the Visual Analog Scale (VAS), the Western Ontario and McMaster Universities Osteoarthritis Index (WOMAC), and joint space narrowing (JSN). The detailed characteristics of the included studies are summarized in Tables 1, 2.

**Table 1:** Characteristics of included meta-analyses.

Study ID	Population	Intervention	Control	Outcomes	Method of analysis	Time span	The number and types of studies related to glucosamine included	Quality assessment tool(s)
Liu et al. (25)	Adults with knee osteoarthritis	Glucosamine	Placebo	Pain, physical function, stiffness, structural outcomes, adverse events, analgesic use, quality of life	Meta-analysis	~ 18 April 2017	18 RCTs	Cochrane RoB
Simental-Mendía et al. (26)	Adults with knee osteoarthritis	Glucosamine	Placebo	Pain, physical function, stiffness, global assessment.	Systematic Review and Meta-analysis	through 2017	10 RCTs	Cochrane RoB
Lee et al. (27)	Adults with knee osteoarthritis	Glucosamine sulfate	Placebo	Structural progression, structural changes	Systematic Review and Meta-analysis	~ July 2008	14 RCTs	Cochrane RoB
Zhu et al. (28)	Adults with knee osteoarthritis	Glucosamine	Placebo	Pain, function, stiffness, adverse events	Systematic Review and Meta-analysis	through 2017	8 RCTs	Cochrane RoB
Vo et al. (6)	Adults with knee osteoarthritis	Glucosamine	Placebo	Pain, function, stiffness, safety outcomes	Systematic Review and Meta-analysis	through 2022	8 RCTs	Cochrane RoB
Park and Kim (29)	Adults with knee osteoarthritis	Glucosamine sulfate	Placebo	Pain, function, structural progression, adverse events	Systematic Review and Meta-analysis	~ 2024	2 RCTs	Cochrane RoB
Ogata et al. (30)	Adults with knee osteoarthritis	Glucosamine	Placebo	Pain, function, stiffness	Systematic Review and Meta-analysis	2003 ~ 2016	10 RCTs	Cochrane RoB
Wu et al. (31)	Adults with knee osteoarthritis	Glucosamine sulfate/hydrochloride	Placebo	Pain, function	Systematic Review and Meta-analysis	~ March 2012.	15 RCTs	Jadad score
Rabade et al (32)	Adults with knee osteoarthritis	Glucosamine sulfate	Placebo	Pain, function, stiffness, structural progression, safety outcomes	Systematic Review and Meta-analysis	~ 2023	4 RCTs	Cochrane RoB
Runhaar et al. (33)	Adults with knee osteoarthritis	Glucosamine	Placebo	Pain, function	Systematic Review and Meta-analysis	~ 2015	4 RCTs	Cochrane RoB
Eriksen et al. (34)	Adults with knee osteoarthritis	Glucosamine	Placebo	Pain	Systematic Review and Meta-analysis	~ 2013	25 RCTs	Not Reported
Gregori et al. (35)	Adults with knee osteoarthritis	Glucosamine sulfate/hydrochloride	Placebo	Pain, Function, structural progression	Systematic Review and Meta-analysis	~ June 30, 2018	3 RCTs	Cochrane RoB
Honvo et al. (36)	Adults with knee osteoarthritis	Glucosamine sulfate	Placebo	Safety outcomes	Systematic Review and Meta-analysis	~ May 31, 2017	8 RCTs	Cochrane RoB
Richy et al. (37)	Adults with knee osteoarthritis	Glucosamine sulfate	Placebo	Pain, function, structural progression	Meta-analysis	1980 ~ 2002	6 RCTs	Jadad score

**Table 2:** Characteristics of included systematic reviews.

Study ID	Population	Intervention	Control	Outcomes	Method of analysis	Time span	The number and types of studies related to glucosamine included	Quality assessment tool(s)
Gallagher et al. (38)	Adults with knee osteoarthritis	Glucosamine sulfate/hydrochloride	Placebo	Structural joint integrity measures, pain outcomes	Systematic review	~ June 2013	3 RCTs	Jadad score
Dostrovsky et al. (39)	Adults with knee osteoarthritis	Glucosamine	Placebo	All glucose metabolism measures except random or single fasting glucose	Systematic review	1950 to February 2009	11 RCTs	Jadad score
Black et al. (40)	Adults with knee osteoarthritis	Glucosamine	Placebo	Disease progression outcomes, pain, physical function, long-term need for knee arthroplasty, adverse events and quality of life	Systematic review	July 2008	3 RCTs	CRD methods
Zhu et al. (28)	Adults with knee and/or hip OA	Glucosamine (various forms), chondroitin, acetaminophen, celecoxib	Placebo	Pain, function and adverse events	Network meta-analysis	October 23, 2017	61 RCTs	Cochrane Risk of Bias Tool
Charlesworth et al (41)	Adults with knee osteoarthritis	NSAIDs, paracetamol, glucosamine, chondroitin, intra-articular corticosteroids	Placebo	safety outcomes and harms, symptom outcomes, structural outcomes, progression to total knee arthroplasty, and serious adverse events.	Systematic review	July 2017	3 RCTs	Cochrane Risk of Bias Tool
Morais et al. (42)	Adults with knee osteoarthritis	Nutritional interventions including dietary modification (Mediterranean diet, weight reduction), omega-3 and omega-6 fatty acid modulation, antioxidants (vitamins A, C, E), vitamins D and K, and minerals (magnesium, selenium, zinc)	Placebo	Pain, inflammation, physical function, mobility, quality of life, structural progression, oxidative stress markers, inflammatory cytokines, and metabolic parameters.	Systematic review	March 2025	—	AMSTAR 2 and GRADE

One included network meta-analysis (28) was summarized descriptively in this table because quantitative data specific to glucosamine could not be extracted for robust variance estimation. A further publication initially retrieved as a systematic review (4) was determined on full-text assessment to be an umbrella review of glucosamine sulfate; it was therefore removed from the included primary syntheses and used separately as an external reference point for cross-validation rather than being counted among the included studies.

### 3.2 Methodological quality assessment (AMSTAR-2)

Based on the AMSTAR-2 assessment of the 19 included systematic reviews and meta-analyses, one was rated as high quality, three as moderate quality, eight as low quality, and four as critically low quality, with the remaining three reviews awaiting completion of full domain-level appraisal. Overall, the majority of included reviews were rated as low or critically low quality, indicating substantial methodological limitations in the existing evidence base on glucosamine for knee osteoarthritis. Therefore, the findings of the present umbrella review should be interpreted with caution (Table 3).

**Table 3:** Methodological quality assessment of included systematic reviews using AMSTAR-2.

Study ID	1	2	3	4	5	6	7	8	9	10	11	12	13	14	15	16	Overall quality
Eriksen et al. (34)	Y	Y	Y	Y	Y	Y	PY	Y	Y	N	Y	Y	Y	Y	PY	Y	Moderate
Gregori et al. (35)	Y	Y	Y	Y	Y	Y	N	Y	Y	N	Y	Y	Y	Y	N	Y	Low
Liu et al. (25)	Y	Y	Y	Y	Y	Y	N	Y	Y	N	Y	Y	Y	Y	P Y	Y	Low
Simental-Mendía et al. (26)	Y	N	Y	Y	Y	Y	N	Y	Y	N	Y	N	Y	Y	PY	Y	Low
Lee et al. (27)	Y	N	Y	Y	N	N	N	Y	N	N	Y	N	N	Y	Y	Y	Critically Low
Zhu et al. (28)	Y	N	Y	Y	Y	Y	N	Y	Y	N	Y	Y	Y	Y	PY	Y	Low
Wu et al. (31)	Y	N	Y	Y	Y	Y	N	Y	PY	N	Y	N	Y	Y	Y	Y	Low
Rabade et al (32)	Y	N	Y	Y	Y	Y	N	Y	Y	N	Y	Y	Y	Y	PY	Y	Low
Runhaar et al. (33)	Y	PY	Y	Y	Y	Y	Y	Y	Y	N	Y	Y	Y	Y	PY	Y	Moderate
Honvo et al. (36)	Y	Y	Y	Y	Y	Y	Y	Y	Y	N	Y	Y	Y	Y	Y	Y	High
Richy et al. (37)	Y	N	Y	Y	Y	Y	N	Y	PY	N	Y	N	N	Y	N	Y	Critically Low
Black et al. (40)	Y	N	Y	Y	Y	Y	N	Y	Y	N	N	Y	Y	Y	PY	Y	Low
Gallagher et al. (38)	Y	N	Y	Y	N	N	N	Y	PY	N	N	N	N	Y	N	Y	Critically Low
Dostrovsky et al. (39)	Y	N	Y	Y	Y	Y	N	Y	PY	N	N	Y	Y	Y	N	Y	Low
Morais et al. (42)	Y	N	Y	Y	N	N	N	Y	N	N	N	N	N	Y	N	Y	Critically Low
Charlesworth et al (41)	Y	Y	Y	Y	Y	Y	Y	Y	Y	N	N	Y	Y	Y	PY	Y	Moderate
Vo et al. (6)	NR	NR	NR	NR	NR	NR	NR	NR	NR	NR	NR	NR	NR	NR	NR	NR	Pending appraisal*
Park and Kim (29)	NR	NR	NR	NR	NR	NR	NR	NR	NR	NR	NR	NR	NR	NR	NR	NR	Pending appraisal*
Ogata et al. (30)	NR	NR	NR	NR	NR	NR	NR	NR	NR	NR	NR	NR	NR	NR	NR	NR	Pending appraisal*

Y: Yes, N: No, PY: Partial Yes; NR: Not reported/appraisal pending. *Vo et al. (6), Park and Kim (29), and Ogata et al. (30) were reconciled into this table to match the included studies in Tables 1, 2; their full domain-level AMSTAR-2 appraisal is to be completed by the authors and the overall rating updated accordingly.

1. PICO components included? 2. Protocol registered before review? (CRITICAL). 3. Study designs explained? 4. Comprehensive literature search? (CRITICAL). 5. Study selection in duplicate? 6. Data extraction in duplicate? 7. List of excluded studies provided? (CRITICAL). 8. Included studies described adequately? 9. Risk of bias assessed? (CRITICAL). 10. Funding sources of primary studies reported? 11. Appropriate meta-analytic methods? (CRITICAL). 12. Impact of RoB on results considered? 13. RoB discussed in interpretation? 14. Heterogeneity explained? 15. Publication bias assessed? (CRITICAL). 16. Conflict of interest declared? Overall Quality (High/Moderate/Low/Critically Low).

### 3.3 Summary of pooled effects of glucosamine versus placebo on pain across included meta-analyses

Pain outcomes were reported across multiple meta-analyses comparing glucosamine with placebo, primarily assessed using VAS and WOMAC-based pain measures. As summarized in Table 4, most pooled estimates showed effect sizes favoring glucosamine, particularly for VAS pain outcomes. Several meta-analyses reported statistically significant pain reduction with glucosamine compared with placebo, although the magnitude of effect varied substantially across studies and outcome scales. A large meta-analysis by Eriksen et al. (34), including 25 trials and 3,458 participants, reported a statistically significant reduction in VAS pain favoring glucosamine compared with placebo (SMD − 0.51, 95% CI − 0.72 to −0.30). Similarly, Zhu et al. (28) found a modest but significant improvement in VAS pain across nine studies (SMD − 0.27, 95% CI − 0.45 to −0.09). Notably, heterogeneity was considerable in some analyses, especially those with larger sample sizes or mixed formulations of glucosamine, indicating variability in study populations and methodological approaches (29–32, 34).

**Table 4:** Summary of pooled effects of glucosamine versus placebo on pain across included meta-analyses.

Study ID	Intervention	Control	Outcome	No. of studies	No. of participants (Intervention/Control)	Pooled effect	Heterogeneity	Favors
							(I2)	
Zhu et al. (28)	Glucosamine	Placebo	VAS	9	2000	−0.27 (95% CI: −0.45, −0.09)	—	Glucosamine
Vo et al. (6)	Glucosamine	Placebo	VAS	6	584/584	−7.41 (95% CI: −14.3, −0.51)	—	Glucosamine
Park and Kim (29)	Glucosamine	Placebo	VAS	2	59/54	−0.82 (95% CI: −1.28, −0.36)	29%	Glucosamine
Ogata et al. (30)	Glucosamine	Placebo	VAS	10	282/281	−0.19 (95% CI: −0.36, −0.03)	0%	Glucosamine
Wu et al. (31)	Glucosamine sulfate	Placebo	VAS	15	—	−0.22 (95% CI: −0.48, 0.04)	82.3%	Glucosamine
Wu et al. (31)	Glucosamine hydrochloride	Placebo	VAS	4	—	−0.16 (95% CI: −0.34, 0.01)	0%	Glucosamine
Rabade et al (32)	Glucosamine	Placebo	VAS	3	75/80	−5.10 (95% CI: −14.5, 4.30)	99%	—
Runhaar et al. (33)	Glucosamine	Placebo	VAS	4	1,403	0.91 (95% CI: −1.91, 3.75)	—	—
Eriksen et al. (34)	Glucosamine	Placebo	VAS	25	3,458	−0.51 (95% CI: −0.72, −0.30)	88%	Glucosamine
Gregori et al. (35)–1	Glucosamine sulfate	Placebo	VAS	2	207	−4.07 (95% CI: −6.99, −1.18)	—	Glucosamine
Gregori et al. (35)–2	Glucosamine hydrochloride	Placebo	VAS	3	325	−2.54 (95% CI: −6.59, 1.55)	—	—
Liu et al. (25)	Glucosamine	Placebo	WOMAC	4	939	−0.17 (95% CI: −0.30, −0.04)	0%	Glucosamine
Vo et al. (6)	Glucosamine	Placebo	WOMAC	8	520/507	−2.27 (95% CI: −5.21, 0.66)	—	—
Park and Kim (29)	Glucosamine	Placebo	WOMAC	2	146/134	0.26 (95% CI: −0.15, 0.66)	56%	—
Ogata et al. (30)	Glucosamine	Placebo	WOMAC	6	654/650	−0.06 (95% CI: −0.17, 0.05)	50%	—
Rabade et al (32)	Glucosamine	Placebo	WOMAC	4	354/374	−0.10 (95% CI: −0.25, 0.05)	7%	—
Runhaar et al. (33)–1	Glucosamine hydrochloride	Placebo	WOMAC	3	1,058	0.78 (95% CI: −4.33, 5.89)	—	—
Runhaar et al. (33)–2	Glucosamine sulfate	Placebo	WOMAC	2	345	−0.38 (95% CI: −3.67, 2.90)	—	—
Lee et al. (27)	Glucosamine	Placebo	WOMAC Pain	14	2,845	−0.105 (95% CI: −0.254, 0.045)	—	—
Vo et al. (6)	Glucosamine	Placebo	WOMAC Pain	8	520/507	−0.04 (95% CI: −0.13, 0.06)	—	—
Ogata et al. (30)	Glucosamine	Placebo	WOMAC Pain	7	815/805	−0.04 (95% CI: −0.13, 0.06)	51%	—

Where reviews reported glucosamine salt formulations separately, a descriptive pattern was apparent in the direction (though not always the statistical significance) of effect. For VAS pain, Wu et al. (31)reported a pooled SMD of −0.22 (95% CI − 0.48 to 0.04) for glucosamine sulfate versus −0.16 (95% CI − 0.34 to 0.01) for glucosamine hydrochloride, and Gregori et al. (35) reported a larger VAS reduction for sulfate (−4.07, 95% CI − 6.99 to −1.18) than for hydrochloride (−2.54, 95% CI − 6.59 to 1.55), the latter crossing the null. A comparable formulation-related gradient was seen for structural outcomes, where Gregori et al. (35) reported a significant slowing of joint space narrowing for glucosamine sulfate (SMD − 0.42, 95% CI − 0.65 to −0.19) but not for hydrochloride (SMD − 0.07, 95% CI − 0.26 to 0.11) (35). These descriptive comparisons are consistent with prior reports that trial quality and brand/formulation, rather than glucosamine per se, may explain part of the observed inconsistency across trials, and that patented crystalline glucosamine sulfate may behave differently from over-the-counter hydrochloride preparations (34, 43). Because formulation was inconsistently reported at the review level and the relevant strata were derived from overlapping primary trials, these subgroup comparisons were not pooled in the robust variance estimation model and should be regarded as hypothesis-generating.

### 3.4 Function and structural outcomes: pooled effects of glucosamine versus placebo

Functional and structural outcomes were evaluated using WOMAC subscales, the Lequesne Index, and JSN change across the included meta-analyses (Table 5). Overall, results for functional outcomes were inconsistent. For example, Lee et al. (27) reported a statistically significant improvement in WOMAC function favoring glucosamine (SMD − 0.31, 95% CI − 0.61 to −0.002), whereas several other meta-analyses, including those by Vo et al. (6) and Ogata et al. (30), showed non-significant effects. Consistent with this, most review-level estimates for WOMAC function and WOMAC stiffness in Table 5 had confidence intervals that crossed the null (for example, Ogata et al. (30) SMD − 0.07, 95% CI − 0.17 to 0.03; Rabade et al. (32) SMD − 0.11, 95% CI − 0.25 to 0.04), indicating that any functional benefit, where present, was small in magnitude. Results for the Lequesne Index were mixed but more often favored glucosamine, with significant effects reported by Park et al. (29), Rabade et al. (32), and for the hydrochloride stratum of Wu et al. (31). For structural outcomes, the joint space narrowing estimates summarized in Table 5 were consistently in favor of glucosamine and frequently statistically significant (Lee et al. (27) SMD − 0.43, 95% CI − 0.63 to −0.24; Richy et al. (37) SMD − 0.41, 95% CI − 0.60 to −0.21; Rabade et al. (32) SMD − 0.29, 95% CI − 0.42 to −0.15), although heterogeneity was high in some analyses (I^2^ up to 85%) and the effect was confined to the sulfate formulation in the one review reporting both salts (35).

**Table 5:** Summary of pooled effects of glucosamine versus placebo on function across included meta-analyses.

Study ID	Intervention	Control	Outcome	No. of studies	No. of participants (Intervention/Control)	Pooled Effect	Heterogeneity	Favors
							(I2)	
Lee et al. (27)	Glucosamine	Placebo	WOMAC Stiffness	11	—	−0.126 (95% CI: −0.264, 0.012)	—	—
Vo et al. (6)	Glucosamine	Placebo	WOMAC Stiffness	8	520/507	−0.07 (95% CI: −0.17, 0.03)	—	—
Liu et al. (25)	Glucosamine	Placebo	WOMAC Function	4	939	−0.17 (95% CI: −0.34, 0.00)	41%	—
Lee et al. (27)	Glucosamine	Placebo	WOMAC Function	8	—	−0.31 (95% CI: −0.61, −0.002)	—	Glucosamine
Vo et al. (6)	Glucosamine	Placebo	WOMAC Function	8	520/507	−0.30 (95% CI: −0.82, 0.21)	—	—
Ogata et al. (30)	Glucosamine	Placebo	WOMAC Function	7	802/790	−0.07 (95% CI: −0.17, 0.03)	37%	—
Rabade et al (32)	Glucosamine	Placebo	WOMAC Function	4	364/347	−0.11 (95% CI: −0.25, 0.04)	0%	—
Runhaar et al. (33)–1	Glucosamine hydrochloride	Placebo	WOMAC Function	3	1,058	0.85 (95% CI: −4.43, 6.13)	—	—
Runhaar et al. (33)–2	Glucosamine sulfate	Placebo	WOMAC Function	2	345	0.62 (95% CI: −2.29, 3.52)	—	—
Park and Kim (29)	Glucosamine	Placebo	Lequesne Index	1	106/104	−0.32 (95% CI: −0.59, −0.04)	—	Glucosamine
Wu et al. (31)–1	Glucosamine sulfate	Placebo	Lequesne Index	3	—	−0.55 (95% CI: −1.22, 0.11)	92.9%	—
Wu et al. (31)–2	Glucosamine hydrochloride	Placebo	Lequesne Index	2	—	−0.36 (95% CI: −0.56, −0.17)	86.2%	Glucosamine
Rabade et al (32)	Glucosamine	Placebo	Lequesne Index	3	246/264	−1.15 (95% CI: −1.79, −0.51)	0%	Glucosamine
Lee et al. (27)	Glucosamine	Placebo	JSN change	2	159/160	−0.432 (95% CI: −0.628, −0.235)	—	Glucosamine
Rabade et al (32)	Glucosamine	Placebo	JSN change	3	286/277	−0.29 (95% CI: −0.42, −0.15)	85%	Glucosamine
Gregori et al. (35)–1	Glucosamine sulfate	Placebo	JSN change	2	207	−0.42 (95% CI: −0.65, −0.19)	—	Glucosamine
Gregori et al. (35)–2	Glucosamine hydrochloride	Placebo	JSN change	3	325	−0.07 (95% CI: −0.26, 0.11)	—	Glucosamine
Richy et al. (37)	Glucosamine	Placebo	JSN change	2	414	−0.41 (95% CI: −0.60, −0.21)	—	Glucosamine

### 3.5 Adverse events: pooled effects of glucosamine versus placebo

Adverse events associated with glucosamine were evaluated in two meta-analyses comparing glucosamine with placebo (Table 6). Both analyses reported no statistically significant differences in the risk of adverse events between the glucosamine and placebo groups. Specifically, Lee et al. (27) reported a pooled risk ratio (RR) of 0.90 (95% CI 0.66 to 1.23), and Honvo et al. (36) reported a similar estimate (RR 0.96, 95% CI 0.66 to 1.41), indicating comparable safety profiles between the two groups.

**Table 6:** Summary of pooled effects of glucosamine versus placebo on adverse events across included meta-analyses.

Study ID	Intervention	Control	Outcome	No. of studies	No. of participants (intervention/control)	Pooled effect	Heterogeneity	Favors
							(I2)	
Lee et al. (27)	Glucosamine	Placebo	Adverse events (RR)	8	—	0.90 (95% CI: 0.66, 1.23)	—	—
Honvo et al. (36)	Glucosamine	Placebo	Adverse events (RR)	8	764/712	0.96 (95% CI: 0.66, 1.41)	29.8%	—

### 3.6 Primary outcomes based on robust variance estimation

**Figure 2:**
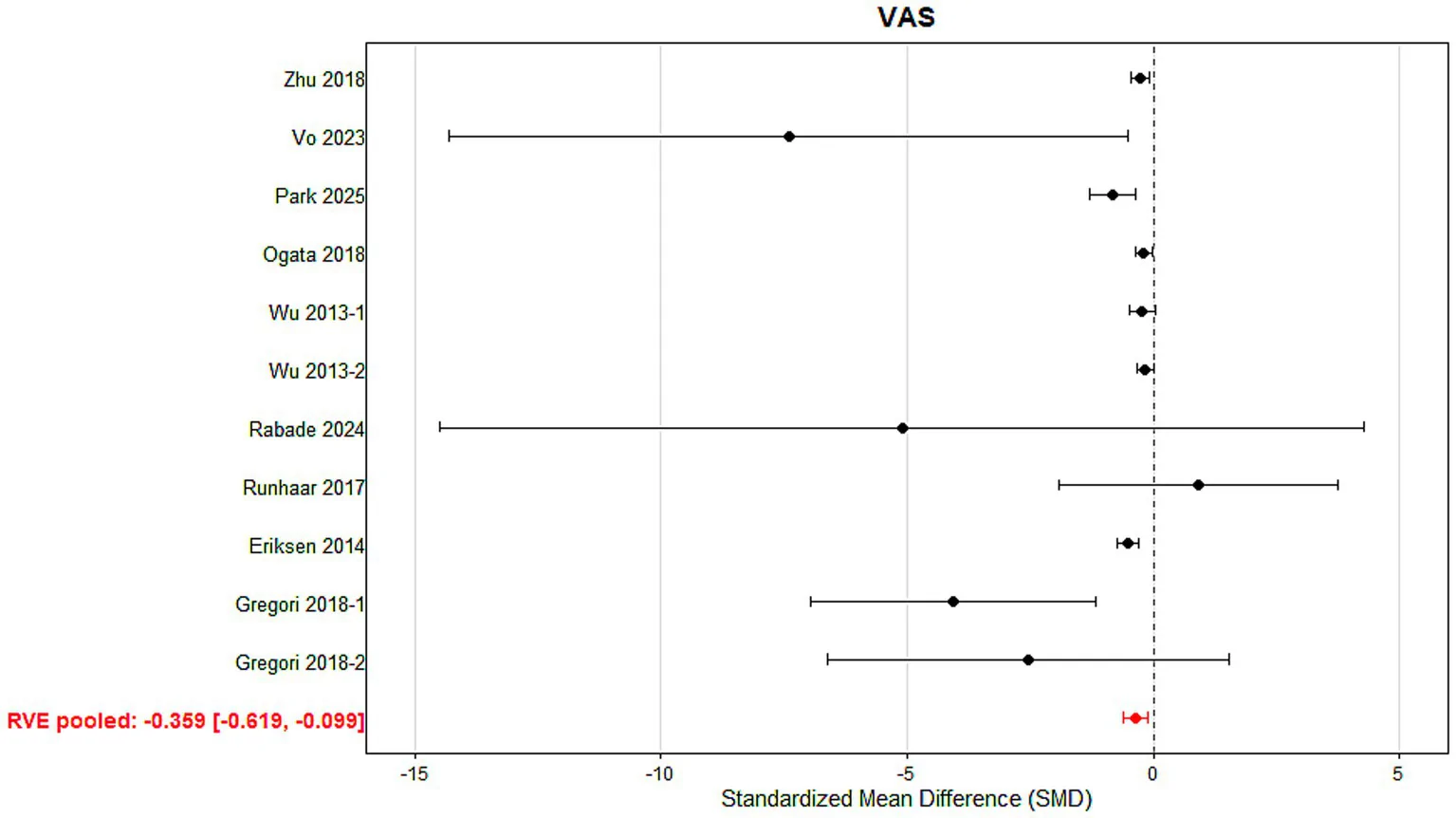
RVE-based pooled effect for VAS.

**Figure 3:**
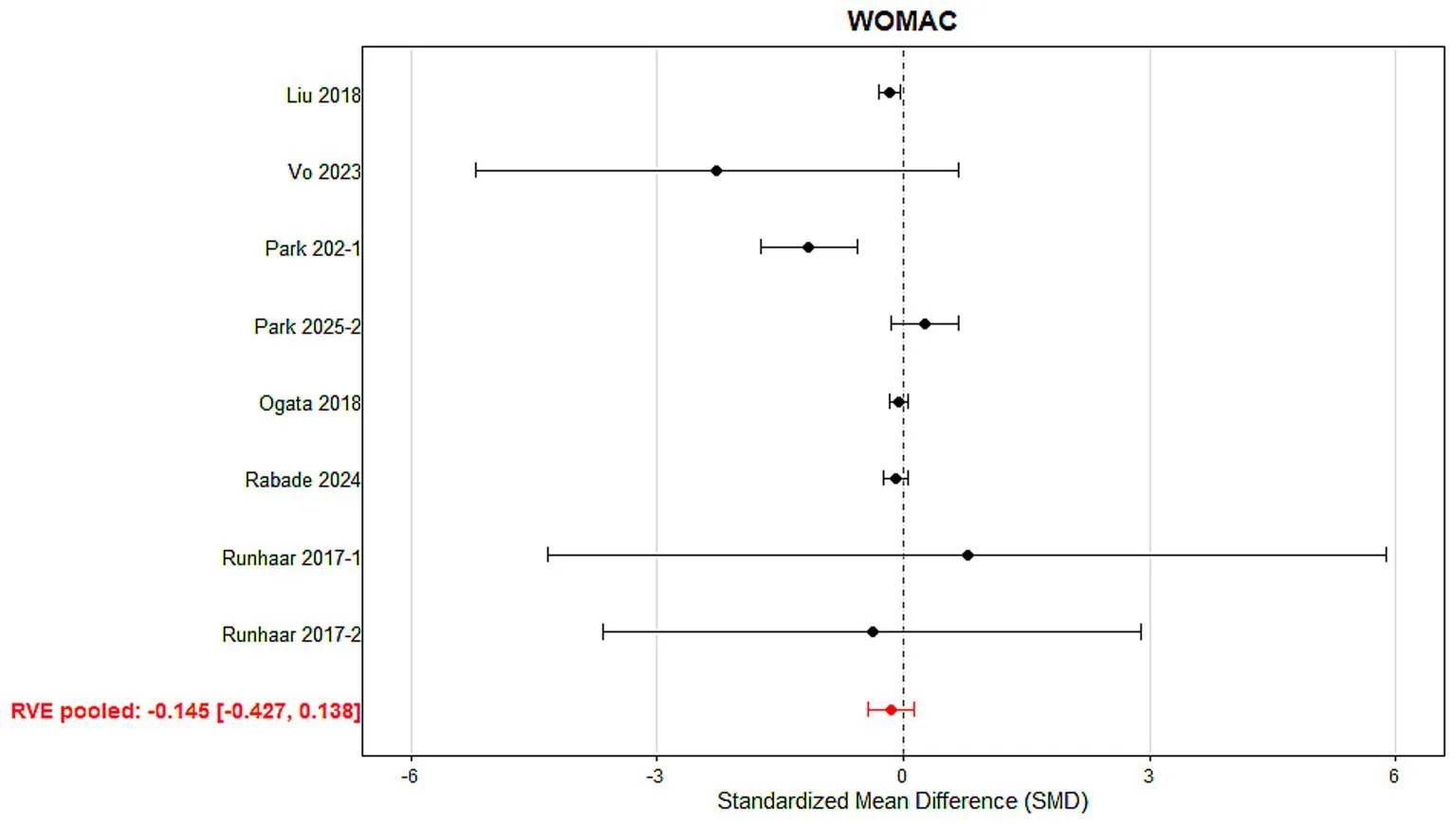
RVE-based pooled effect for WOMAC.

**Figure 4:**
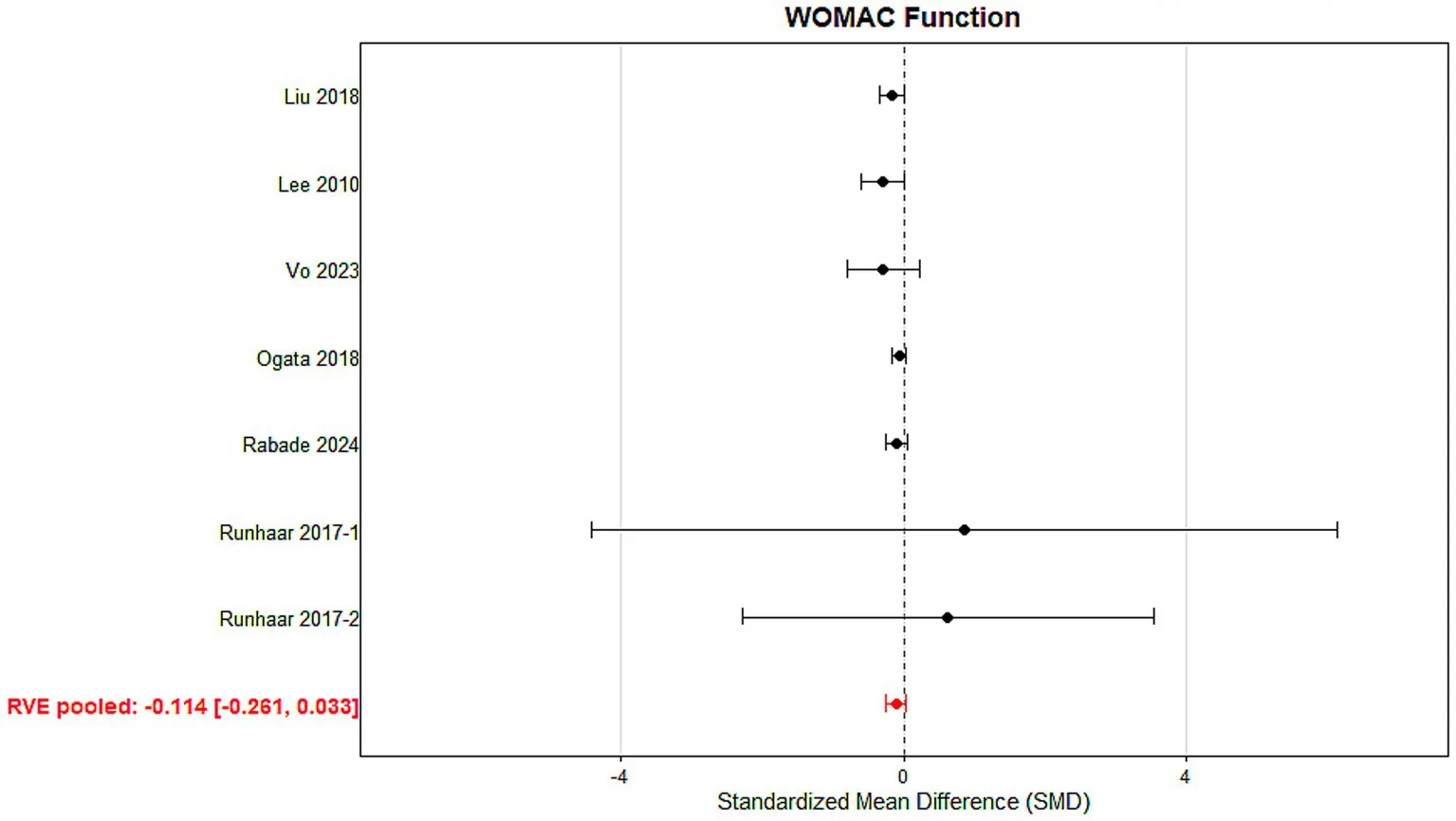
RVE-based pooled effect for WOMAC function.

**Figure 5:**
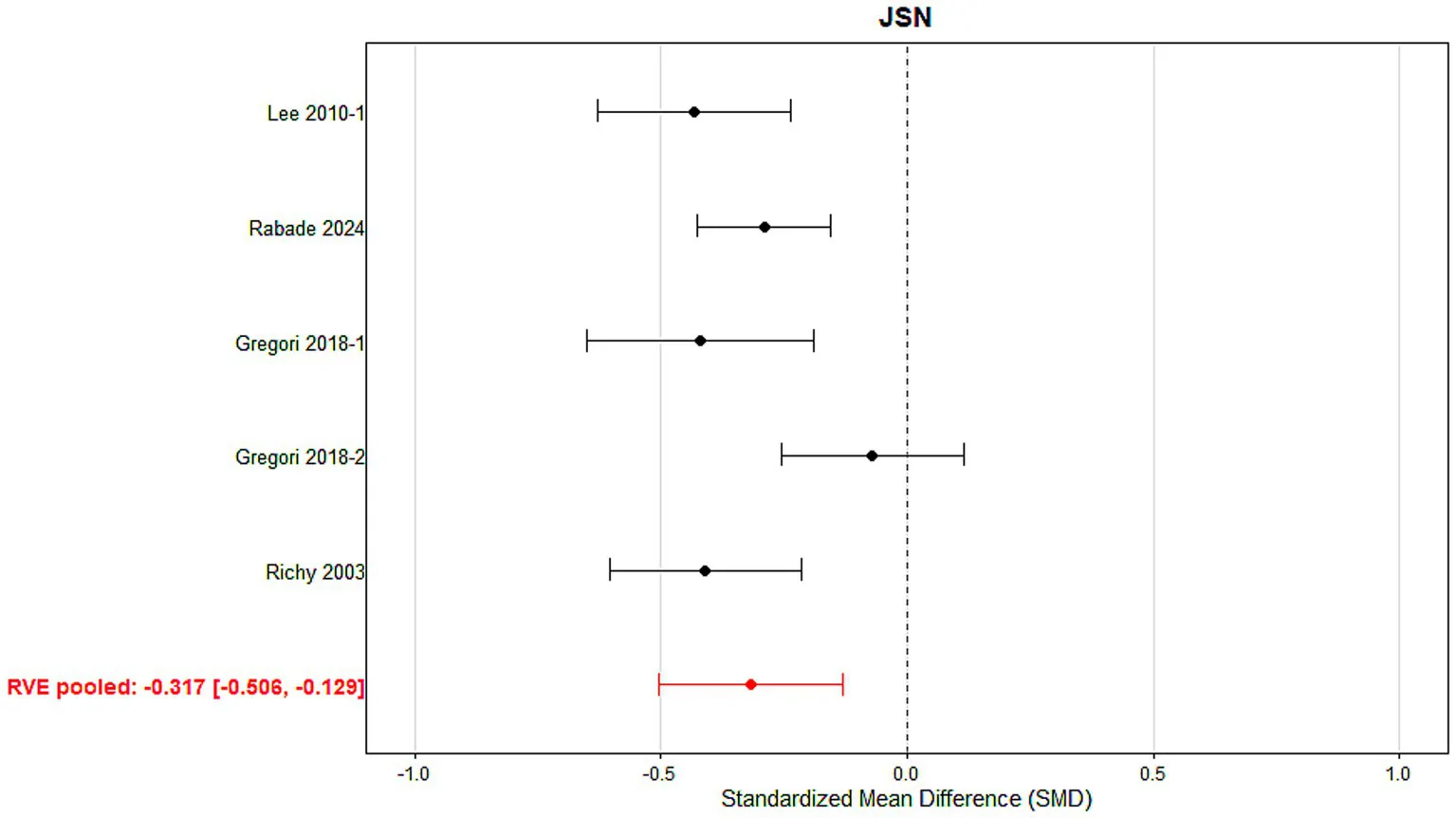
RVE-based pooled effect for JSN.

Table 7 summarizes the primary outcomes synthesized using robust variance estimation (RVE). After accounting for dependence among effect sizes, significant pooled effects were observed for VAS pain and joint space narrowing (JSN). Specifically, glucosamine was associated with a small reduction in VAS pain (SMD − 0.36, 95% CI − 0.62 to −0.10; *p* = 0.019) and a significant slowing of JSN progression (SMD − 0.32, 95% CI − 0.51 to −0.13; *p* = 0.0096). In contrast, pooled effects for WOMAC total score (SMD − 0.14, 95% CI − 0.43 to 0.14; *p* = 0.209) and WOMAC function (SMD − 0.11, 95% CI − 0.26 to 0.03; *p* = 0.081) did not reach statistical significance, although both outcomes showed trends toward improvement. To aid clinical interpretation, these effect sizes should be weighed against established benchmarks: by the conventional Cohen criteria all of the pooled estimates fall within the “small” range (|SMD| ≤ 0.36), and the VAS pain estimate lies below the SMD of approximately 0.37 that has been proposed as the minimal clinically important difference for pain in osteoarthritis trials, corresponding to roughly 9 mm on a 100-mm scale (20–23). Accordingly, while the pain and structural effects were statistically significant, their magnitude is at or below the threshold generally regarded as clinically important, and they should be interpreted as modest at the population level rather than as evidence of a substantial individual treatment benefit. The RVE-based pooled effect estimates for each outcome are further illustrated in Figures 2–5.

**Table 7:** Summary of primary outcomes based on robust variance estimation (RVE).

Outcome	Pooled effect size, SMD (95% CI)	*p* value	Statistical significance	Interpretation
VAS pain	−0.36 (−0.62 to −0.10)	0.019	Significant	Statistically significant but small reduction (small effect, at/below MCID; see footnote)
WOMAC total score	−0.14 (−0.43 to 0.14)	0.209	Not significant	Trend toward improvement, but not statistically significant
WOMAC function	−0.11 (−0.26 to 0.03)	0.081	Not significant	Trend toward functional improvement, but not statistically significant
JSN (joint space narrowing)	−0.32 (−0.51 to −0.13)	0.0096	Significant	Statistically significant but small slowing of joint space narrowing; signal driven mainly by long-duration sulfate trials

SMD, standardized mean difference; CI, confidence interval; VAS, visual analog scale; WOMAC, Western Ontario and McMaster Universities Osteoarthritis Index; JSN, joint space narrowing; MCID, minimal clinically important difference. Note: Effect sizes are interpreted using Cohen benchmarks (approximately 0.2, 0.5, and 0.8 for small, moderate, and large effects). For pain in osteoarthritis trials, a standardized effect of approximately 0.37 (about 9 mm on a 100-mm VAS) has been proposed as the minimal clinically important difference; the VAS pain estimate observed here (SMD −0.36) lies at or just below this threshold, so the significant effects should be regarded as small and of uncertain individual clinical importance.

## 4 Discussion

Based on the robust variance estimation (RVE)–based umbrella meta-analysis, the effects of glucosamine varied across different outcome domains. Overall, glucosamine demonstrated a small but statistically significant benefit for pain relief assessed by VAS and for structural progression measured by joint space narrowing (JSN). In contrast, the pooled effects for overall functional outcomes assessed by the WOMAC total score and for WOMAC function did not reach statistical significance, showing only non-significant trends toward improvement. As detailed below, even the statistically significant effects were small in magnitude and at or below accepted thresholds of clinical importance, so the overall picture is one of limited clinical relevance rather than a clear treatment benefit.

Among pain-related outcomes, VAS showed more consistent and robust improvements compared with WOMAC-based pain or total scores. This discrepancy may be partly explained by differences in outcome sensitivity. As a unidimensional measure, VAS is more sensitive to changes in pain intensity, whereas WOMAC is a multidimensional composite instrument incorporating pain, stiffness, and function, in which the pooled effect on pain may be diluted by other domains. Glucosamine produced a small but statistically significant reduction in knee pain on visual analog scales, whereas WOMAC-based pain and function scores showed only non-significant trends. This discrepancy is consistent with prior observations that VAS measures can detect subtle treatment effects more readily than multi-item scales. For example, da Costa et al. found that VAS scores yielded larger effect sizes than WOMAC pain subscales in most trials (44), and Simental-Mendía et al. (45) likewise reported a clear glucosamine benefit on VAS pain but no meaningful improvement in total WOMAC scores. In line with this, Ogata et al. noted a marginal VAS pain improvement with glucosamine while the WOMAC function change was small and non-significant (30). Thus, our pain-outcome pattern likely reflects differences in instrument sensitivity rather than a contradiction in efficacy.

Interpretation of function outcomes should be cautious. We saw only a trend toward better WOMAC function with glucosamine, without meeting statistical significance. This mirrors other analyses: some meta-analyses (Lee et al.) found a modest WOMAC function gain, but most high-quality reviews find little or no functional benefit (27). Overall, the weight of evidence suggests glucosamine’s impact on mobility or stiffness is minimal. Balancing benefits and harms, even the modest symptomatic relief we found must be weighed against safety. Notably, no meta-analysis has shown more adverse events with glucosamine than placebo; pooled risk ratios for any side effect are essentially null (4). Our umbrella findings of equivalent AE rates are in line with other reviews concluding that glucosamine is well tolerated and carries no excess safety risk (4).

When compared with prior research, our results largely support recent reviews finding only small benefits. For instance, Vo et al. (6) also reported significant pain reduction with glucosamine and found no serious adverse events. Our estimated pain effect (SMD–0.36) is similar to those earlier syntheses, though effect sizes vary by outcome measure. An unexpected nuance is that we observed a significant slowing of radiographic joint space narrowing. Long-term trials have hinted at this possibility: in a 3-year RCT, Pavelka et al. (46) showed virtually no cartilage loss on glucosamine vs. clear narrowing on placebo. Some meta-analyses likewise suggest glucosamine may slow progression when used for ≥3 years (46). Thus, our structural finding adds new perspective to the debate, even as symptom improvements remain modest. In summary, glucosamine appears to provide a small benefit for pain with no added harm, a conclusion consistent with much of the literature.

For structural outcomes, joint space narrowing (JSN) demonstrated a statistically significant improvement, indicating a potential role of glucosamine in slowing structural degeneration. The partial discordance between structural and symptomatic outcomes is consistent with the well-recognized phenomenon of symptom–structure dissociation in osteoarthritis, in which radiographic change and patient-reported symptoms are frequently discordant (47). Notably, the structural signal in the literature derives chiefly from long-duration trials: the two pivotal 3-year randomized studies of prescription crystalline glucosamine sulfate reported retardation of joint space narrowing relative to placebo, and an 8-year observational follow-up of their participants suggested fewer subsequent total knee replacements, whereas shorter trials generally did not detect a structural effect (8, 46, 48). This duration dependence implies that any disease-modifying potential, if real, would require sustained treatment over years rather than weeks to months. These findings therefore provide supportive, though indirect, evidence for a possible structural effect of glucosamine that should be confirmed in dedicated long-term trials.

These findings also have socioeconomic and patient-centered implications that merit emphasis given the very large scale of glucosamine use. Glucosamine is among the most widely consumed dietary supplements for osteoarthritis and is most often purchased over the counter as an out-of-pocket expense rather than being reimbursed; industry estimates of the global glucosamine market vary by source and scope but consistently run into the hundreds of millions to several billion US dollars annually, implying substantial aggregate consumer spending. When set against effect sizes that are statistically significant but at or below the minimal clinically important difference, the cost-effectiveness of routine glucosamine use becomes an important consideration. This concern is reinforced by formal economic evaluation: a United Kingdom health technology assessment concluded that the cost-effectiveness of glucosamine and chondroitin for knee osteoarthritis could not be demonstrated with confidence given the uncertainty in the underlying clinical evidence (40). From a patient-centered perspective, the outcomes that matter most to people with knee OA—sustained pain relief, improved mobility and physical function, preserved independence, and avoidance of joint replacement—were precisely those for which our synthesis found either no significant effect (WOMAC function and total score) or only small, duration-dependent, and indirect signals (structural progression). Patients and clinicians weighing glucosamine should therefore balance its favorable safety profile and low risk against a modest and possibly subclinical expected benefit and a recurring personal financial cost, and these trade-offs should be communicated transparently in shared decision-making.

A major strength of this study is the application of robust variance estimation to synthesize effect sizes across multiple systematic reviews and meta-analyses, thereby accounting for statistical dependence arising from overlapping primary studies. Notably, after applying the more conservative RVE correction, significant effects for pain and structural outcomes remained, which enhances the robustness and credibility of the findings. Several limitations should be acknowledged. First, the methodological quality of the included reviews was generally low, with only one review rated high and the majority rated low or critically low on AMSTAR-2, so the certainty of the pooled estimates is correspondingly limited. Second, although robust variance estimation accounts for statistical dependence arising from overlapping primary trials, the included reviews shared many of the same underlying randomized trials, so the body of evidence is narrower than the number of reviews suggests, and the synthesis cannot overcome biases present in the original trials. Third, dose, regimen, treatment duration, and salt formulation (sulfate versus hydrochloride) were inconsistently reported at the review level, which precluded formal dose- and duration-stratified pooling; our formulation comparisons are therefore descriptive and hypothesis-generating rather than confirmatory. Fourth, the statistically significant effects we observed for VAS pain and joint space narrowing were small and at or below accepted minimal clinically important difference thresholds, so their clinical relevance is uncertain. Finally, the restriction to English-language, peer-reviewed publications may introduce language and publication bias. Future research should prioritize adequately powered, low-risk-of-bias randomized trials with prespecified long-term structural endpoints, standardized and clearly reported formulations and dosing, head-to-head comparisons of glucosamine sulfate versus hydrochloride, and concurrent collection of patient-centered and cost-effectiveness outcomes to better clarify the place of glucosamine in knee OA management.

## 5 Conclusion

In this umbrella review, we evaluated the efficacy and safety of glucosamine in knee osteoarthritis using robust variance estimation to account for overlap among reviews. Glucosamine was associated with a statistically significant but small reduction in knee pain on VAS and with a statistically significant slowing of joint space narrowing, whereas functional outcomes (WOMAC total and function) did not reach statistical significance. Adverse event rates were comparable to placebo. Importantly, the magnitude of the significant effects was at or below established minimal clinically important difference thresholds, so their relevance to the individual patient is uncertain, and the structural signal appears confined to long-duration treatment with the sulfate formulation. These findings are further tempered by the generally low methodological quality of the included reviews, extensive overlap of their primary trials, inconsistent reporting of dose and formulation, and substantial heterogeneity. In conclusion, glucosamine appears to confer at most a small symptomatic benefit and a possible long-term structural effect in knee OA, with a reassuring safety profile but limited demonstrable clinical importance; given that it is predominantly an out-of-pocket therapy, these modest effects should be weighed against its cost in shared decision-making, and adequately powered, low-risk-of-bias trials reporting patient-centered and economic outcomes are needed before routine use can be recommended.

## Data Availability

The original contributions presented in the study are included in the article/Supplementary material, further inquiries can be directed to the corresponding authors.
