# Supplementary material for: Glucosamine for knee osteoarthritis: an umbrella review
Source: Front Nutr. 2026 Aug 11;13:1806413. doi: 10.3389/fnut.2026.1806413 (PMC13503159; doi:10.3389/fnut.2026.1806413)
Supplement: Supplementary file 1 [file Table_1.docx]

**Supplementary Table 1. Search strategies**

**Pubmed (searching date: Nov 25, 2025)**

| **Category** | **Step** | **Terms** | **Result** |
| --- | --- | --- | --- |
| Knee Osteoarthritides | #1 | "Osteoarthritis, Knee"[Mesh] OR “Knee Osteoarthritides” [tiab] OR “Knee Osteoarthritis”[tiab] OR “Osteoarthritis of Knee”[tiab] OR “Osteoarthritis of the Knee”[tiab] | 40482 |
|  | #2 | "Knee"[Mesh] OR “Knee”[tiab] OR "Knee Joint"[Mesh] OR “Knee Joint”[tiab] OR “Knee Joints”[tiab] OR “Superior Tibiofibular Joint”[tiab] OR “Superior Tibiofibular Joints”[tiab] | 224287 |
|  | #3 | "Osteoarthritis"[Mesh] OR “Osteoarthritis”[tiab] OR “Osteoarthritides”[tiab] OR “Degenerative Arthritides”[tiab] OR “Degenerative Arthritis”[tiab] OR “Osteoarthrosis”[tiab] OR “Osteoarthroses”[tiab] OR “Osteoarthrosis Deformans”[tiab] OR “Arthrosis”[tiab] OR “Arthroses”[tiab] | 137615 |
|  | #4 | #2 AND #3 | 57672 |
|  | #5 | #1 OR #4 | 59760 |
| Glucosamine | #6 | "Glucosamine"[Mesh] OR “Glucosamine”[tiab] OR “2-Amino-2-Deoxyglucose”[tiab] OR “2 Amino 2 Deoxyglucose”[tiab] OR “Glucosamine Sulfate”[tiab] OR “Dona”[tiab] OR “Dona S”[tiab] OR “Hespercorbin”[tiab] OR “Xicil”[tiab] OR “SYSADOA”[tiab] OR “Symptomatic slow-acting drugs in osteoarthritis”[tiab] | 25847 |
| Terms for study designs | #7 | "Systematic Review"[pt] OR "Systematic Reviews as Topic"[Mesh] OR "Meta-Analysis"[pt] OR "Meta-Analysis as Topic"[Mesh] OR "Network Meta-Analysis"[pt] OR "Network Meta-Analysis as Topic"[Mesh] OR "Scoping Review"[pt] OR "Scoping Review as Topic"[Mesh] OR "Guideline"[pt] OR "Guidelines as Topic"[Mesh] OR "Practice Guideline"[pt] OR "Evidence-Based Practice"[Mesh] OR "Consensus"[Mesh] OR "Consensus Development Conference, NIH"[pt] OR "Consensus Development Conference"[pt] OR "Consensus Development Conferences, NIH as Topic"[Mesh] OR "Consensus Development Conferences as Topic"[Mesh] OR "systematic review"[tiab] OR "meta-analysis"[tiab] OR "umbrella review"[tiab] OR "scoping review"[tiab] OR "overview"[tiab] OR guideline[tiab] OR guide[tiab] OR guidance[tiab] OR practice[tiab] OR recommendation[tiab] OR consensus[tiab] | 608053 |
| Combined | #8 | #5 AND #6 AND #7 | 89 |

**Embase (searching date: Nov 25, 2025)**

| **Category** | **Step** |  |  |
| --- | --- | --- | --- |
| Knee Osteoarthritides | #1 | 'knee osteoarthritis'/exp  OR 'knee osteoarthritides':ti,ab,kw  OR 'knee osteoarthritis':ti,ab,kw  OR 'osteoarthritis of knee':ti,ab,kw  OR 'osteoarthritis of the knee':ti,ab,kw | 61870 |
|  | #2 | 'knee'/exp  OR 'knee':ti,ab,kw  OR 'knee joint'/exp  OR 'knee joint':ti,ab,kw  OR 'knee joints':ti,ab,kw  OR 'superior tibiofibular joint':ti,ab,kw  OR 'superior tibiofibular joints':ti,ab,kw | 300976 |
|  | #3 | 'osteoarthritis'/exp  OR 'osteoarthritis':ti,ab,kw  OR 'osteoarthritides':ti,ab,kw  OR 'degenerative arthritides':ti,ab,kw  OR 'degenerative arthritis':ti,ab,kw  OR 'osteoarthrosis':ti,ab,kw  OR 'osteoarthroses':ti,ab,kw  OR 'osteoarthrosis deformans':ti,ab,kw  OR 'arthrosis':ti,ab,kw  OR 'arthroses':ti,ab,kw | 233273 |
|  | #4 | #2 AND #3 | 89101 |
|  | #5 | #1 OR #4 | 95276 |
| Glucosamine | #6 | 'glucosamine'/exp  OR 'glucosamine':ti,ab,kw  OR '2-amino-2-deoxyglucose':ti,ab,kw  OR '2 amino 2 deoxyglucose':ti,ab,kw  OR 'glucosamine sulfate':ti,ab,kw  OR 'dona':ti,ab,kw  OR 'dona s':ti,ab,kw  OR 'hespercorbin':ti,ab,kw  OR 'xicil':ti,ab,kw  OR 'sysadoa':ti,ab,kw  OR 'symptomatic slow-acting drugs in osteoarthritis':ti,ab,kw | 33886 |
| Terms for study designs | #7 | (  'meta analysis'/exp  OR 'systematic review'/exp  OR 'meta analysis':ti,ab,kw  OR 'meta-analyses':ti,ab,kw  OR 'meta-analysis':ti,ab,kw  OR 'systematic review':ti,ab,kw  OR 'systematic reviews':ti,ab,kw  OR 'pooled analysis':ti,ab,kw  OR 'quantitative review':ti,ab,kw  OR 'umbrella review':ti,ab,kw  OR 'overview of reviews':ti,ab,kw  OR 'meta synthesis':ti,ab,kw  ) | 889213 |
| Combined | #8 | #5 AND #6 AND #7 | 241 |

**Cochrane Library (searching date: Nov 25, 2025)**

| **Category** | **Terms** | **Result** |
| --- | --- | --- |
| Knee Osteoarthritides | #1 MeSH descriptor: [Osteoarthritis] explode all trees  #2 MeSH descriptor: [Knee] explode all trees  #3 #1 AND #2  #4 ("knee osteoarthritis"  OR "knee osteoarthritides"  OR "osteoarthritis of knee"  OR "osteoarthritis of the knee"):ti,ab,kw  #5 ("knee" OR "knee joint" OR "knee joints"  OR "superior tibiofibular joint"  OR "superior tibiofibular joints"):ti,ab,kw  #6 ("osteoarthritis" OR "osteoarthritides"  OR "degenerative arthritides"  OR "degenerative arthritis"  OR "osteoarthrosis"  OR "osteoarthroses"  OR "osteoarthrosis deformans"  OR "arthrosis"  OR "arthroses"):ti,ab,kw  #7 #3 OR #4 OR (#5 AND #6) | 20656 |
| Glucosamine | #8 MeSH descriptor: [Glucosamine] explode all trees  #9 ("glucosamine"  OR "2-amino-2-deoxyglucose"  OR "2 amino 2 deoxyglucose"  OR "glucosamine sulfate"  OR "dona"  OR "dona s"  OR "hespercorbin"  OR "xicil"  OR "sysadoa"  OR "symptomatic slow-acting drugs in osteoarthritis"):ti,ab,kw  #10 #8 OR #9 | 1022 |
| Terms for study designs | #11 MeSH descriptor: [Meta-Analysis as Topic] explode all trees  #12 MeSH descriptor: [Systematic Reviews as Topic] explode all trees  #13 ("meta analysis"  OR "meta-analysis"  OR "meta-analyses"  OR "systematic review"  OR "systematic reviews"  OR "umbrella review"  OR "overview of reviews"):ti,ab,kw)  #14 #11 OR #12 OR #13 | 35232 |
| Combined | #15 #7 AND #10 AND #14 | 34 |

**EBSCOhost (searching date: Nov 25, 2025)**

| **Category** | **Step** | **Terms** | **Result** |
| --- | --- | --- | --- |
| Knee Osteoarthritides | #1 | (MH "Osteoarthritis+") AND (MH "Knee+")  OR TI("knee osteoarthritis" OR "knee osteoarthritides"  OR "osteoarthritis of knee" OR "osteoarthritis of the knee")  OR AB("knee osteoarthritis" OR "knee osteoarthritides"  OR "osteoarthritis of knee" OR "osteoarthritis of the knee")  OR TX(("knee" OR "knee joint" OR "knee joints"  OR "superior tibiofibular joint" OR "superior tibiofibular joints")  AND  ("osteoarthritis" OR "osteoarthritides" OR "degenerative arthritides"  OR "degenerative arthritis" OR "osteoarthrosis" OR "osteoarthroses"  OR "osteoarthrosis deformans" OR "arthrosis" OR "arthroses") | 14 |
| Glucosamine | #2 | MH "Glucosamine+"  OR TI("glucosamine" OR "2-amino-2-deoxyglucose" OR "2 amino 2 deoxyglucose"  OR "glucosamine sulfate" OR "dona" OR "dona s" OR "hespercorbin"  OR "xicil" OR "sysadoa" OR "symptomatic slow-acting drugs in osteoarthritis")  OR AB("glucosamine" OR "2-amino-2-deoxyglucose" OR "2 amino 2 deoxyglucose"  OR "glucosamine sulfate" OR "dona" OR "dona s" OR "hespercorbin"  OR "xicil" OR "sysadoa" OR "symptomatic slow-acting drugs in osteoarthritis")  OR TX("glucosamine" OR "2-amino-2-deoxyglucose" OR "2 amino 2 deoxyglucose"  OR "glucosamine sulfate" OR "dona" OR "dona s" OR "hespercorbin"  OR "xicil" OR "sysadoa" OR "symptomatic slow-acting drugs in osteoarthritis") | 266 |
| Terms for study designs | #3 | MH "Meta-Analysis"  OR MH "Meta-Analysis as Topic"  OR MH "Systematic Review"  OR MH "Systematic Reviews as Topic"  OR TI("meta analysis" OR "meta-analysis" OR "meta-analyses"  OR "systematic review" OR "systematic reviews"  OR "umbrella review" OR "overview of reviews")  OR AB("meta analysis" OR "meta-analysis" OR "meta-analyses"  OR "systematic review" OR "systematic reviews"  OR "umbrella review" OR "overview of reviews")  OR TX("meta analysis" OR "meta-analysis" OR "meta-analyses"  OR "systematic review" OR "systematic reviews"  OR "umbrella review" OR "overview of reviews") | 14806 |
| Combined | #4 | #1 AND #2 AND #3 | 3 |
